# Effect of calcium carbonate precipitate derived from sugar beet industry on the properties of poly (vinyl chloride) compounds

**DOI:** 10.1038/s41598-026-57058-4

**Published:** 2026-06-16

**Authors:** Mohamed Abdelhamed, Wagih Abdel Alim Sadik, Abdel-Ghaffar M. EL-Demerdash, Essam El-Rafey

**Affiliations:** 1 Materials Science Department, Institute of Graduate Studies and Research, Alexandria University, Alexandria, Egypt; 2 Egyptian Petrochemicals Company, Alexandria, Egypt

**Keywords:** Polyvinyl Chloride (PVC), Precipitated Calcium Carbonate (PCC) waste, Mechanical Properties, Thermal properties, Engineering, Environmental sciences, Materials science

## Abstract

The incorporation of precipitated calcium carbonate (PCC) waste from the sugar beet industry significantly altered the properties of the PVC matrix. Thermal stability improved markedly, evidenced by a reduction in weight loss (240–360 °C) from 58.88% (neat PVC) to 37% (40 wt% PCC). The flexural strength reached a maximum at 5 wt% PCC, representing a 35% improvement over the unfilled PVC. Conversely, this loading adversely affected elongation at break, tensile strength, and impact strength, reducing them by 71.57%, 10.84%, and 17.11%, respectively. Physical properties, such as specific gravity and water absorption, increased slightly with the PCC content. SEM analysis confirmed excellent PCC dispersion at lower loadings, which transitioned to particle agglomeration and void formation at higher concentrations, explaining the mechanical properties trends.

## Introduction

Sugar has long been a widely used commodity in the human diet, serving as a source of energy, a sweetener, and a preservative. Chemically known as sucrose, sugar is a type of saccharide that is quickly broken down into fructose and glucose in the human body by the enzyme sucrose^1^. The sugar industry is divided into two main branches: cane sugar and beet sugar^2^. Recently, in 2024/2025, worldwide sugar consumption was about 177 million metric tons (MMT)^3^. Around 27.5% of the world’s sugar is produced from beets; the remainder is from sugar cane. The Russian Federation has the highest sugar beet production worldwide (27.65%). The US, Turkey, Ukraine, China, and Egypt are the remaining five nations that produce the most sugar beets^4^. Egypt’s beet production in the 2018/2019 season reached approximately 12,247,170 tons (62.2% of the country’s sugar production)^5^.

The carbonization process remains the basic clarification process in sugar beet refineries. This process uses calcium carbonate as the main refining agent during the liming stage. Carbon dioxide is gradually injected into raw beet juice and lime water (a suspension of lime milk and calcium hydroxide), resulting in the coagulation of non-sugar components and the formation of calcium carbonate (CC) precipitates, which are then converted to precipitated calcium carbonate (PCC) waste^6^.

PCC is produced in significant quantities and leads to various issues, including environmental pollution, as dried PCC can be easily dispersed by wind. Wind disperses waste particles throughout the entire region, covering any water surfaces. Health issues arise when wind carries particles, posing risks to nearby residents. Additionally, land concerns remain as waste accumulates within the plant’s area. The unpleasant smell of wet PCC and the significant disposal costs that sugar-producing companies face when managing this waste^7^.

PCC is produced by most sugar industries in Egypt and is derived from sugar beet production and refining, accounting for almost 4% of the weight of the sugar beets^8^. This means that approximately 490,000 tons of PCC waste is generated annually, and its disposal is difficult. The disposal process is so expensive that these companies either dispose of PCC in confined or open waste yards or use it for non-scientific landfilling, resulting in environmental pollution, water contamination, and soil contamination. Several approaches have been implemented to utilize PCC, including adding it to animal feed mixtures and using it in cement bricks in the construction industry^4^.

On the other hand, in addition to being one of the most adaptable polymers, PVC is also one of the most well-known and commonly utilized polymers. According to data, global PVC production was 31 million tons per year in 2001 and is projected to reach 59.72 million tons per year by 2030^9^. PVC is commonly used in various kinds of fields, such as construction and the automobile industry, pipelines and cables, and household products, due to its strength, durability, long-lastingness, and versatility. PVC has a service life of over ten years in the construction industry^10^.

The two main categories of PVC are rigid and flexible. The rigid one is utilized in the fields of automobiles, pipelines, health care, building, and construction, while the flexible one is applied in the production of cables, wires, films, tubes, sheets, and bottles^11^. Neat PVC needs some chemical additives to enhance its processability and suitability for applications. Plasticizers, fillers, impact modifiers, lubricants (both internal and external), and color agents are the most used additives in PVC compounding^12^.

One of the most common inorganic mineral functional fillers used in plastic is calcium carbonate^13^. Ground calcium carbonate (GCC) has attracted increasing attention and experienced significant development owing to its straightforward manufacturing process, eco-friendliness, and comparatively lower production costs than those of precipitated calcium carbonate (PCC)^14,15^.

Traditionally, GCC has been used to reduce costs, improve melt viscosity, and enhance the modulus of plastic compounds due to its small surface area and geometric features. Nonetheless, some mechanical characteristics either remained the same or decreased in certain cases. Recent reports indicate that the properties of PVC compounds filled with GCC are significantly affected by particle size and shape, as well as the filler content^16–20^. In recent years, it has become necessary to use such waste as fillers, which can become an alternative source of raw materials and limit the depletion of non-renewable natural resources^21^, and also to overcome the problems that have been produced from the production of PCC. This paper studied a novel approach to modify thermal, mechanical, and physical properties of PVC compounds by incorporating PCC derived from sugar beet industry waste. Using this industrial waste offers a sustainable, cost-effective alternative.

## Experimental

### Materials

Polyvinyl chloride (PVC), suspension grade with K-value 67 with bulk density of 0.542 g/ml, inherent viscosity (IV) of 0.92 dL/gm, Mass Loss (wt%) 0.056, porosity (DOP) 0.22 ml/gm, average particle size (A.P.S) 157 micron and residual vinyl chloride monomer content (R-VCM) 0.62 ppm, in the form of PVC powder. A lead-based one-pack system, under the trade name Akropan 11084-4 FX, was used. Stearic acid (SA), CH_3_ (CH_2_)_16_ COOH, was used as an external lubricant in the formation of a PVC compound. The Egyptian Petrochemicals Company (EPC), located in Alexandria, Egypt, provided all of these chemicals. After 48 h of drying at 105 ± 3 °C, PCC was screened using a 100-mesh screen (140 microns). The calcium carbonate content in PCC, determined by volumetric methods, was 81.3%^22^. PCC’s moisture content was 9.8%. The bulk density of PCC was 0.743 g/ml. The dioctyl Phthalate (DOP) absorption was 54.2 g/100 g. The experiments were conducted at the EPC Polymer Laboratory in Alexandria, Egypt.

### Characterization of PCC

SEM (JEOL-JSM-IT200, Japan) was employed to measure particle size and characterize the shape of PCC. It was gold-coated to increase surface conductivity prior to observation using an SPI-MODULE ion sputtering device with an acceleration voltage of 20 kV^23^.

Dynamic light scattering (DLS), in which a laser beam passes through a solution containing suspended particles, is used by the Zetasizer (ZS) to quantify particle size. The particles scatter the laser, producing constructive or destructive interference. Due to the Brownian motion of suspended particles, the strength of the interference signals varies; autocorrelation functions connect this motion-based variation to particle size^24^. The particle size distribution of PCC was determined by DLS using a Zetasizer Nano (Malvern Instruments Ltd., UK). PCC powder was dispersed in water by sonication in an ultrasonic bath at 25 °C for 60 s, and the suspension was analyzed directly. To determine the average particle diameter, five measurements were taken of the sample. The test was carried out at the polymer laboratory in the City of Scientific Research, Alexandria. Egypt.

The elemental composition and possible oxides in PCC were determined using an Energy-Dispersive X-ray detector (EDX; JEOL-JSM-IT200, Japan) equipped with advanced vacuum systems.

FTIR spectroscopy was carried out for PCC. A sample was prepared as discs by adding potassium bromide (KBr), and the discs were analyzed over the wavenumber range 350–4400 cm^− 1^ on a PerkinElmer Spectrum BX (USA). The test was conducted at the Institute of Graduate Studies and Research (IGSR) at Alexandria University, Egypt.

### Preparation of PVC compounds

PVC and other additives were mixed with formulation as in Table 1 in a high-intensity mixer model (PLASMEC TRL, Italy) (2000 rpm), at a maximum temperature of 120 ± 3 °C following cooling to 35 ± 3 °C and discharge to polyethylene sacks to create neat PVC compound (S0), the determined PCC and PVC compound amounts were blended in two roll mill machines (Hapro-Machinery-China) at 200 °C with a rolling speed of 20 rpm for 10 min, to create PVC/PCC compound sheets in various weight percents of PCC as recorded in Table 2.

**Table 1:** Recipe of PVC compound.

Function	Type	Phr	%
Resin	PVC	100	96.9
One-pack system	Lead-based (Akropan 11084-4 FX)	3	2.9
External lubricant	Stearic acid	0.2	0.19
Total		103.2	100.00

Phr: Part per hundred resins.

**Table 2:** Compositions of prepared PVC/PCC compounds in (wt%).

Sample	PVC compound (wt%)	PCC (wt%)
S0	100	-------
S5	95	5
S10	90	10
S20	80	20
S40	60	40

A compressing molding machine model (HAPRO-AT-R7026-X-China) was used to produce PVC/PCC compounds (sheet) by adjusting the temperature at 200 ± 5 °C and the pressure at 140 bar to have a sheet having 22 cm length × 22 cm width and about 0.4 ± 0.15 cm thickness. Preparation of study sheets using both a two-roll machine and a compressing molding machine was conducted at the Plastic Technology Center, Alexandria, Egypt. Using conventional hollow die punches, the sheets were prepared into dumbbell specimens in accordance with ASTM-D638-03 for the tensile test, into bar forms in accordance with ASTM-D790-03 for the flexural test, and into notched Izod impact test in accordance with ASTM-D256-06a.

### Characterization of PVC/PCC compounds

For all PVC/PCC compounds, as well as the neat PVC compound, FTIR spectroscopy transmission mode was performed. Every 10 × 10 mm sample with a thickness of about 1 mm was prepared using a pair of scissors. An FTIR spectrometer Alpha II (Bruker Nano GmbH, Berlin, Germany) was used to record the spectra. using a wavenumber range between 350 and 4400 cm^-1^.

SEM model (JEOL-JSM-IT200, Japan) was applied to tensile fractured surfaces of all PVC/PCC compound samples to describe their broken surface morphology.

The PVC/PCC compounds were mechanically tested using the EPC’s standard procedures. The mechanical properties of each compound were evaluated as follows: tensile parallel to the surface and elongation at break were measured in accordance with ASTM D638-03; flexural strength was determined following ASTM D790-03. All these tests were conducted using a universal testing machine (Shimadzu Autograph AG, Japan). Notched Izod impact strength was measured according to ASTM D256-06a using an Izod impact tester (Ceast 6546/000.2 N hammer, Italy). The shore hardness (shore D) test was determined according to ASTM D 2240-00 using a Ceast hardness tester weighing 5 kg.

The samples were soaked in distilled water at 23 ± 2 °C for 312 h to perform the water absorption test in accordance with ASTM-D-570-98. After removing the samples from the water, a fresh, dry towel was used to wipe out every surface. Weighing the specimens was done to the closest 0.0001 g. For every sample, five duplicates were examined. The samples were weighed on an electronic scale at regular intervals to measure the water absorption, and the results were computed as follows in Eq. 1:^25^

1 $${\text{Absorption of water }}\left( \% \right){\text{ }}={\text{ }}\left[ {\left( {{{\mathrm{W}}_{\mathrm{2}}} - {\text{ }}{{\mathrm{W}}_{\mathrm{1}}}} \right)/{\text{ }}{{\mathrm{W}}_{\mathrm{1}}}} \right]{\text{ }} \times {\mathrm{1}}00$$

where (W1) is the sample’s mass before soaking in distilled water, and (W2) is the sample’s mass after soaking.

In accordance with ASTM D 792, a pycnometer was used to measure the specific gravity of each compound. After filling it with water, the pycnometer was immersed in a water bath until its temperature equilibrated with the bath. The weight of the water-filled pycnometer was obtained. 1–5 g of the sample was added after washing and drying, and the specimen’s weight, plus the pycnometer, was recorded. Lastly, the specimen’s weight and the weight of the pycnometer filled with water were noted. Equation 2 was used to get the specific gravity:

2 $$\:\mathrm{S}\mathrm{p}\mathrm{e}\mathrm{c}\mathrm{i}\mathrm{f}\mathrm{i}\mathrm{c}\:\mathrm{g}\mathrm{r}\mathrm{a}\mathrm{v}\mathrm{i}\mathrm{t}\mathrm{y}\:=\:\frac{\mathbf{a}}{(\mathbf{a}+\mathbf{b}-\mathbf{m})}$$

Where (a) is the mass of the specimen in grams, (b) is the mass of the pycnometer filled with water in grams, and (m) is the mass in grams of the pycnometer holding the water-filled specimen.

Thermogravimetric analysis (TGA) was performed according to ASTM D 3850-94-2012 using a PerkinElmer Thermogravimetric Analyzer (SHIMADZU TGA-50 H, Japan). The thermal behavior of the prepared samples (5–10 mg) was measured between 25 and 500 °C under a nitrogen atmosphere at a heating rate of 10 °C/min in a platinum cell.

Thermomechanical Analysis (TMA) was performed in accordance with ASTM E1545 using a Shimadzu Thermomechanical Instrument (TMA-60 H, Shimadzu, Japan) to determine the glass transition temperature (T_g_) of the prepared PVC/PCC compounds^26^. An expansion/compression probe was used, with a load of 5 g. In a nitrogen environment at a flow rate of 30 mL/min, the temperature range for the measurements was 25 to 120 °C, with a heating rate of 5 °C/min. Penetration probes apply a small amount of load. The temperature at which the probe penetrates the sample to a specific depth under a certain load is known as the softening temperature (T_s_). Softening is a result of the T_g_ of amorphous polymers, such as PVC, so the Ts is close to the T_g_ in this case^27^. It is a common practice in many polymer laboratories to report T_s_ for T_g_^28^.

## Results and discussion

### Characterization of PCC

**Fig. 1:**
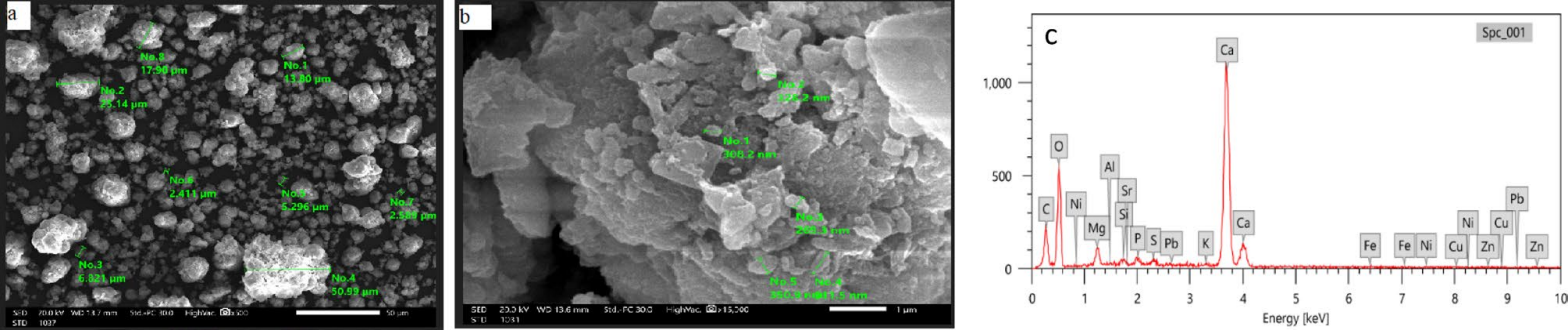
**(a)** SEM micrograph of PCC with magnification 500x, (**b**) with magnification 15000x, (**c**) EDX spectrum of PPC.

Figure 1a displays SEM micrographs showing that individual PCC particles aggregated with a mean particle size of around 25.36 μm. Figures 1b presents higher magnification SEM micrographs, from which the mean size of each primary PCC particle was estimated to be 0.3 ± 0.04 μm. Figures 1c shows the elementary composition of dried PCC particles; the elementary and oxide percentages are recorded in Table 3.

**Table 3:** The elemental and oxide composition of PCC by EDX.

Element or Oxide	Abbreviations	Content wt%	± Standard deviation
Carbon	C	14.45	1.05
Oxygen	O	53.54	1.52
Sodium	Na	0.13	0.01
Magnesium	Mg	1.35	0.50
Aluminum	Al	0.10	0.04
Silicon	Si	0.29	0.09
Phosphorus	P	0.61	0.19
Sulfur	S	0.40	0.05
Potassium	K	0.13	0.05
Calcium	Ca	28.14	2.02
Chromium	Cr	0.03	0.04
Manganese	Mn	0.06	0.28
Iron	Fe	0.13	0.04
Nickel	Ni	0.23	0.00
Cooper	Cu	0.35	0.14
Zinc	Zn	0.29	0.04
Strontium	Sr	0.32	0.17
Lead	Pb	0.51	0.07
Sodium oxide	Na_2_O	0.260	0.020
Magnesium oxide	MgO	3.460	1.247
Aluminum oxide	Al_2_O_3_	0.297	0.107
Silicon oxide	SiO_2_	1.008	0.315
Phosphorus oxide	P_2_O_5_	2.280	0.722
Sulfur oxide	SO_3_	1.615	0.181
Potassium oxide	K_2_O	0.240	0.089
Calcium oxide	CaO	64.420	2.681
Manganese oxide	MnO	0.140	0.000
Ferrous oxide	FeO	0.270	0.070
Nickel oxide	NiO	0.450	0.000
Cooper oxide	CuO	0.700	0.280
Zinc oxide	ZnO	0.565	0.065
Strontium oxide	SrO	0.617	0.331
Lead oxide	PbO	0.885	0.095

Each value is an average of 3 specimens.

**Fig. 2:**
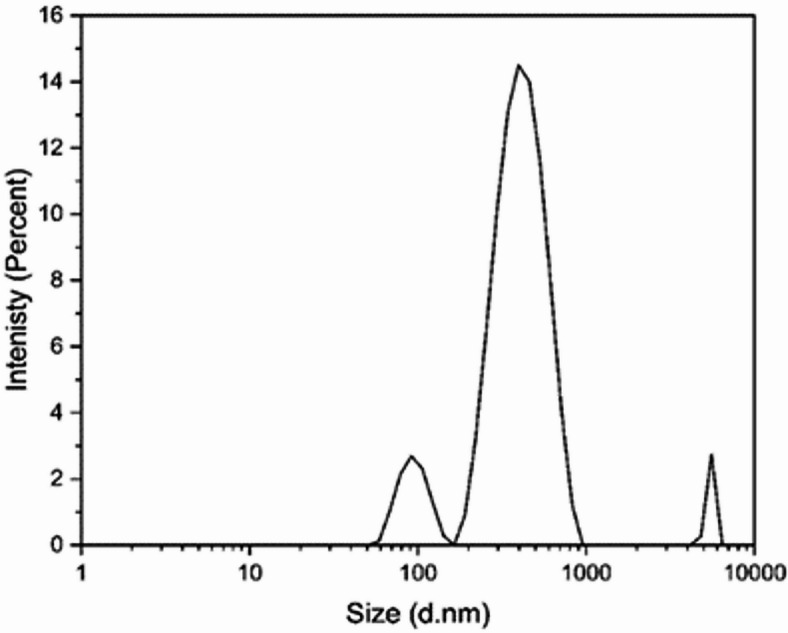
PCC’s particle size distribution.

Figure 2 displays the distribution of PCC’s particle sizes. was 425.7 nm with an intensity of 87.1%, and minor peaks were observed at particle sizes of 94.71 nm and 5493 nm, with intensities of 9.9% and 3%, respectively.

### Characterization of PVC/PCC compounds

#### FTIR

**Fig. 3:**
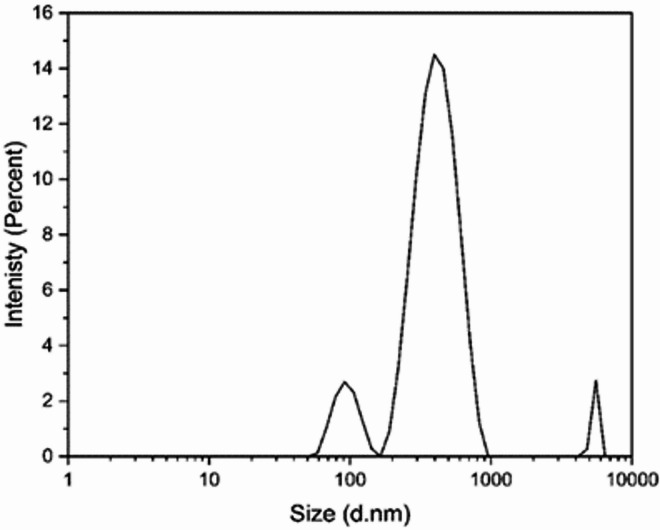
FTIR spectrum of PCC.

FTIR was used to confirm the presence of different functional groups in the components of PCC within PVC compounds. Figures 3 shows the characteristic bands of PCC. The main CaCO_3_ peaks were found at 3420 cm^− 1^, which is attributed to the stretching vibration mode of O-H groups related to the moisture content; The stretching vibrations of C-O were attributed to the peak at 1444 cm^− 1^. For C-O, the peaks at 874 cm^− 1^ and 714 cm^− 1^ were attributed to in-plane and out-of-plane bending vibrations, respectively. Additionally, weaker combination bands are observed at 1798 cm⁻¹ and 2515 cm⁻¹, which are typical features of the calcite crystal structure of CaCO_3_^29,30^.

**Fig. 4:**
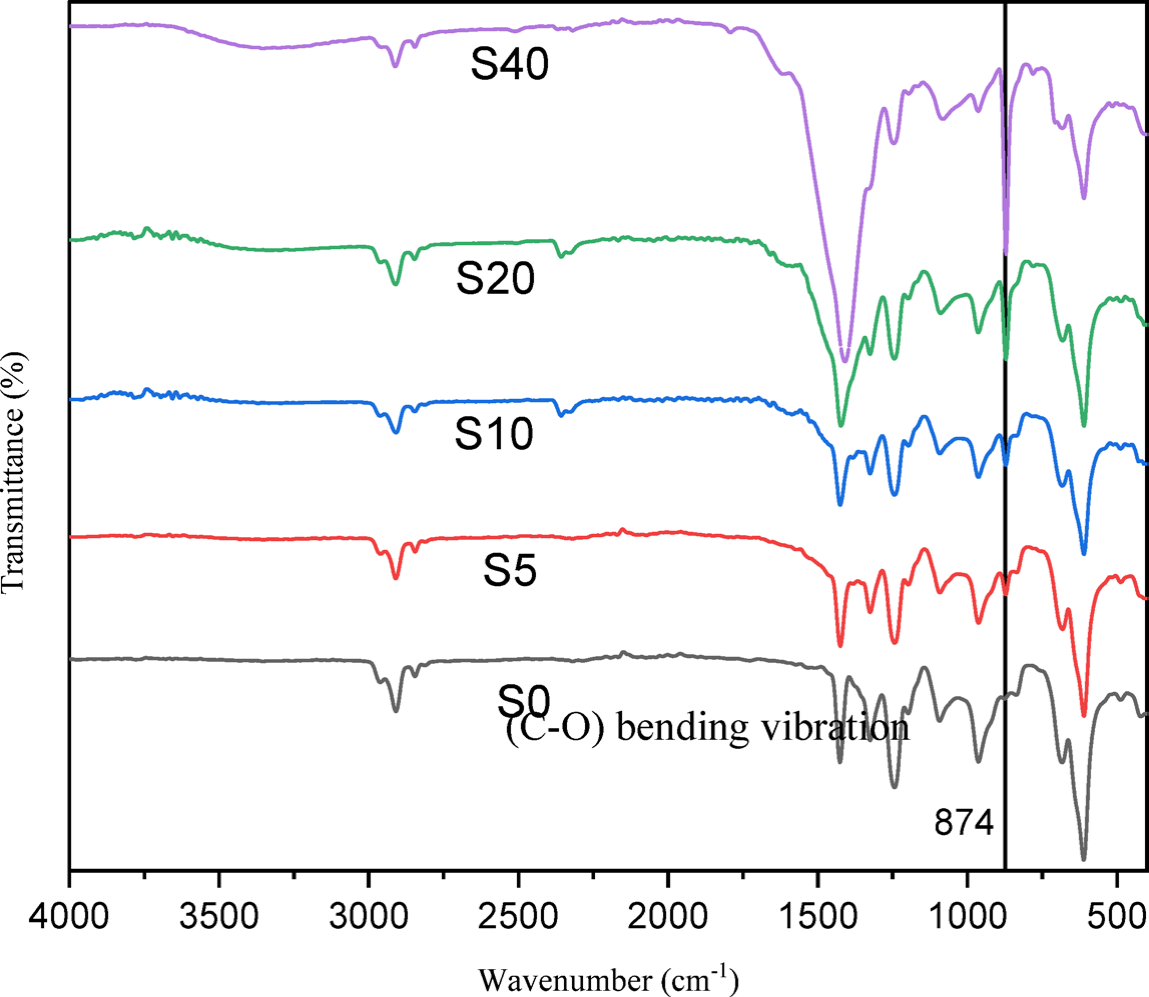
FTIR spectra of S0, S5, S10, S20, and S40.

The FTIR spectra of S0, S5, S10, S20, and S40 are shown in Figure 4. Three areas can be used to categorize the unique bands of PVC compound. The first is known as the (C-Cl) stretching zone, which spans 600–700 cm^− 1^; the second is attributed to (C–C) stretching, which spans 900–1200 cm^− 1^; and the third, which spans 1250–2970 cm^− 1^, is attributed to several (C–H) modes^31,32^. The broad and intense absorption peak of PVC at 1425 cm^− 1^, caused by the wagging (CH_2_) group, overlapped with a significant intensity peak of antisymmetric stretching vibration at 1444 cm^− 1^, as stated by CaCO_3_. Additionally, a novel, strong peak due to the (C-O) bending vibration was observed at 874 cm-1, indicating that PCC is present in PVC compound^19^.

#### SEM

**Fig. 5:**
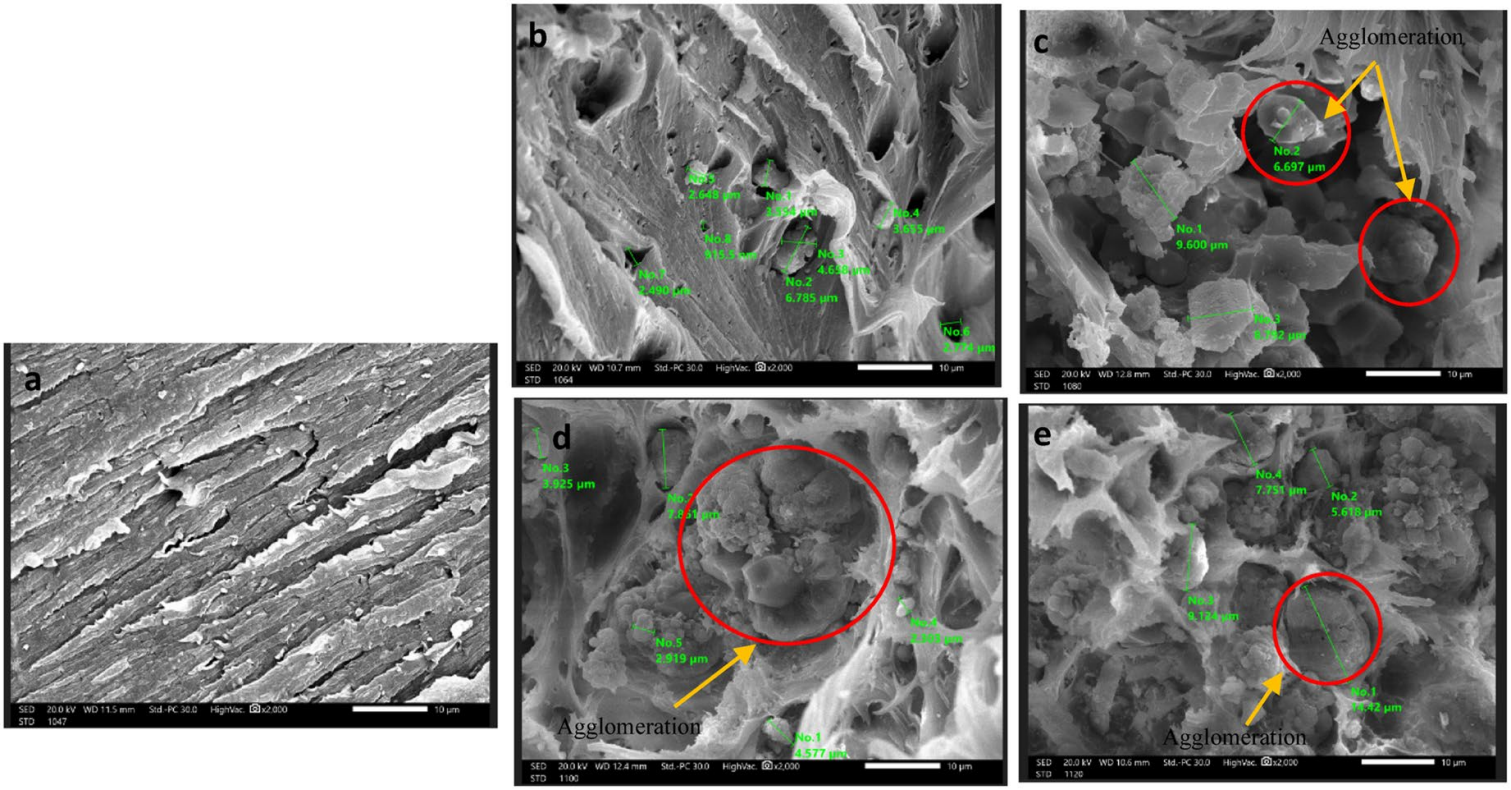
SEM micrographs of (**a**) S0, (**b**) S5, (**c**) S10, (**d**) S20, and (**e**) S40.

SEM images demonstrated the morphology and potential interfacial adhesion between PCC and PVC matrix. The SEM micrograph of the fracture surfaces of the tensile specimens of the neat PVC compound and all PVC/PCC samples is displayed in Figure 5a-e. Neat PVC compound (S0), as shown in Figure 5a, shows a regular, smooth, and homogenous broken surface. From the Figure, it was observed that the presence of voids resulting from debonding at the PCC-PVC matrix interface increased with increasing PCC loading; at PCC loadings above 20 wt%, particle agglomeration occurred. Stress concentration sites occur in certain areas due to poor particle dispersal and larger PCC particle sizes^33,34^. The particle-matrix interface is believed to influence compound characteristics significantly. Strong particle-matrix interface bonds are essential for achieving excellent mechanical properties in compounds^35^.

#### Mechanical properties

**Fig. 6:**
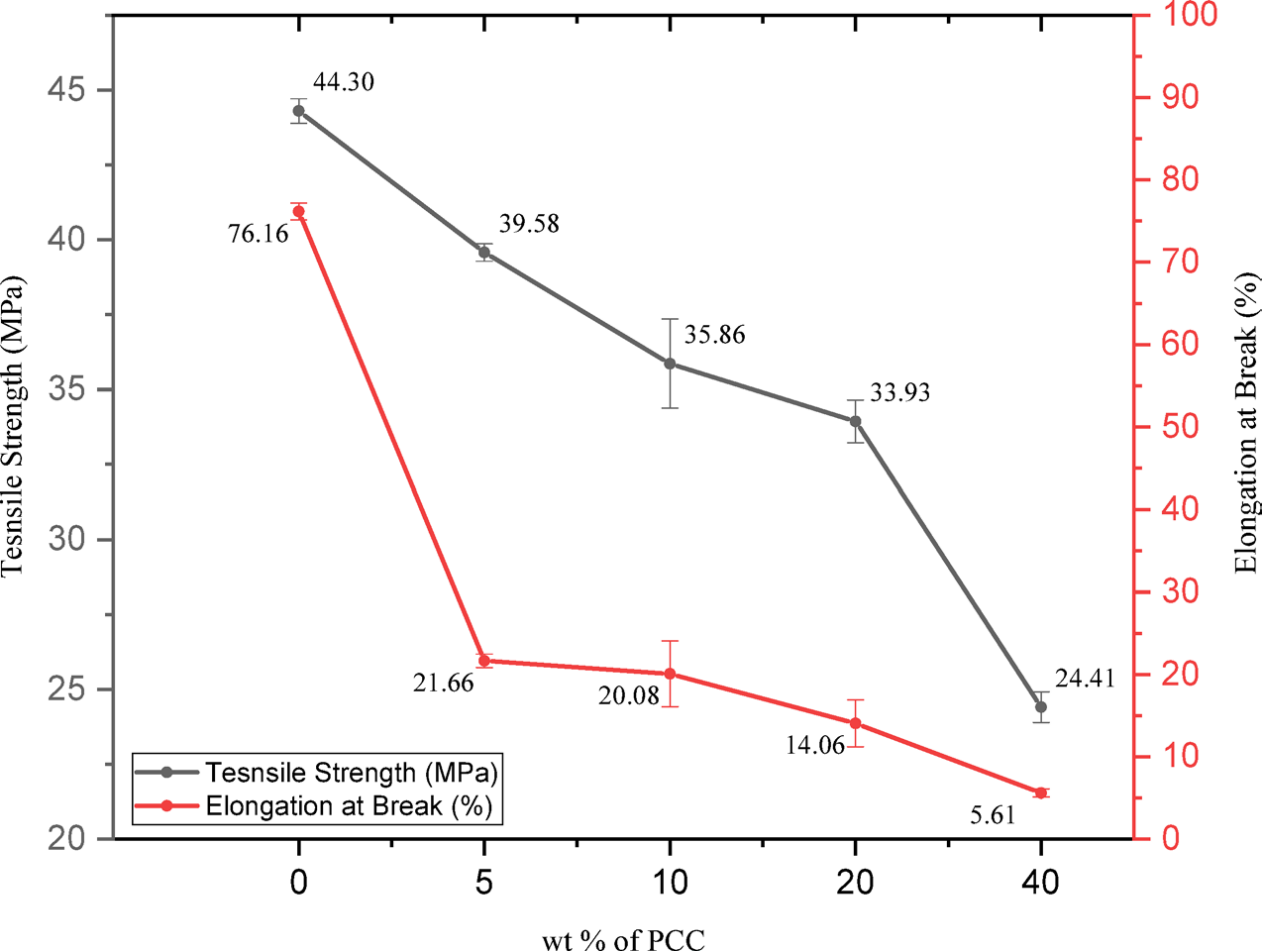
Tensile strength and elongation at break of PVC/PCC compounds.

Tensile strength and elongation at break of the S0, S5, S10, S20, and S40 compounded samples are shown in Figure 6. A considerable decrease in total tensile strength was observed with increasing the amount of PCC in comparison to the neat PVC compound (44.3 MPa). This was related to insufficient bonding between PCC particles and PVC matrix, resulting in interfacial weakness between the filler and the matrix under tensile stress. This behavior agrees with the results of previous studies^36,37^. SEM images of the fracture surface of PVC/PCC compounds at any PCC loading revealed a partly brittle surface with numerous voids caused by particle debonding and fallout. This agrees with the observation that the tensile strength dropped as PCC loading increased. Inadequate particle dispersion and filler agglomeration (leading to higher stress levels in specific areas) resulted in embrittlement of the compounds^38^.

Because the stiff PCC particles act as stress concentrators, while the PVC matrix can elongate, the rigid PCC particles cannot, leading to localized high stress at the PCC-PVC matrix interface that obstructs the movement of PVC molecules. Elongation at break dramatically decreased as the PCC concentration increased in comparison to the neat PVC compound^39^. The compounds’ elasticity decreased due to aggregated filler with increasing PCC loading and dewetting (ineffective stress transfer from the matrix to the filler particles) at the filler-matrix boundary^40^.

**Fig. 7:**
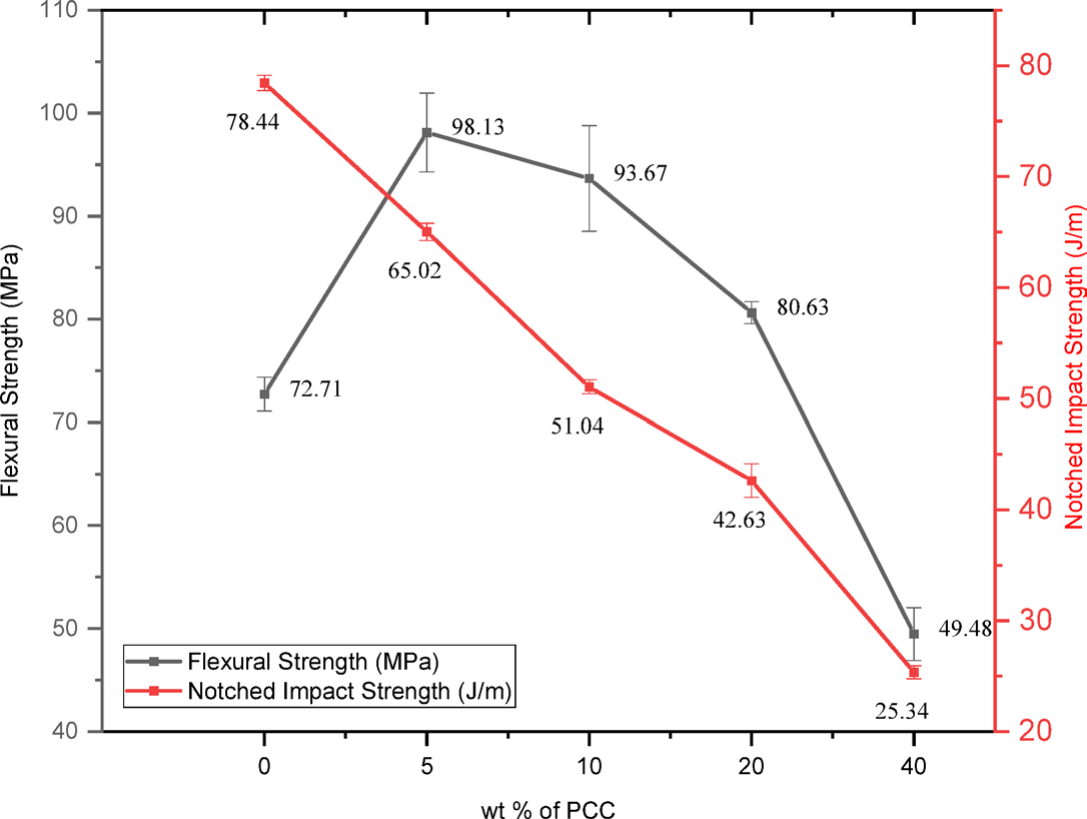
Flexural strength and notched impact strength of PVC/PCC compounds.

The stress at which a material begins to yield in a flexure test is called its flexural strength^41^. The transverse bending test is the most commonly used method, in which a specimen with either a circular or rectangular cross-section is subjected to bending until fracture or yielding occurs, using a three-point loading method. Flexural strength is the maximum stress a material can withstand before yielding. Figures 7 shows the flexural strength of PVC/PCC compounds. Firstly, flexural strength increased when PCC was added at 5 wt% compared with the neat PVC compound, then decreased gradually beyond that level. The flexural strength increased from 72.71 MPa for S0 to 98.31 MPa for S5, then decreased to 93.67 MPa for S10, then decreased to 80.63 MPa for S20, and also kept decreasing down to 49.48 MPa for S40. Generally, the increase in flexural strength with increasing PCC loading is due to several factors. According to the literature, the addition of small PCC particles significantly reduces the micropores and interfaces inside PVC polymer chains^42^. Also, because the compound had to be formed inside the mold, there was an excess of polymer on the surface. This resulted in smooth composite surfaces, preventing cracking of the sample’s top and bottom surfaces during bending^25^. Furthermore, improved stress transfer at the matrix-filler interfaces under bending loads and increased mechanical anchoring of the matrix provided by rough PCC particles may be responsible for the improvement in flexural strength^43,44^. The gradual decrease in flexural strength with increasing PCC loading may be due to PCC particles agglomerating within the PVC matrix at excessive filling levels, resulting in uneven dispersion, brittle regions, and reduced compound strength^45^.

Factors including the toughness characteristics of the reinforcement, the qualities of the matrix-filler interfaces, and the cracked behavior of the polymer matrix influence the impact strength of a compound. Toughness is proportional to a material’s energy absorption capacity during plastic deformation. Because they can withstand only a limited amount of plastic deformation, brittle materials have low toughness^40,46^. Figure 7 shows the notched Izod impact strength of PVC/PCC compounds. It was found that the impact strength decreased with the addition of PCC compared to the neat PVC compound. This may be due to the significant stress immediately transmitted from PVC polymeric matrix to PCC particles. Mineral particles filled with plastic compounds were previously found to have a similar reduction in impact strength^46–48^. PCC particles limit plastic deformation by acting as areas of stress concentration. The material fractures brittlely when the fracture extends into the interfacial regions upon impact.

**Fig. 8:**
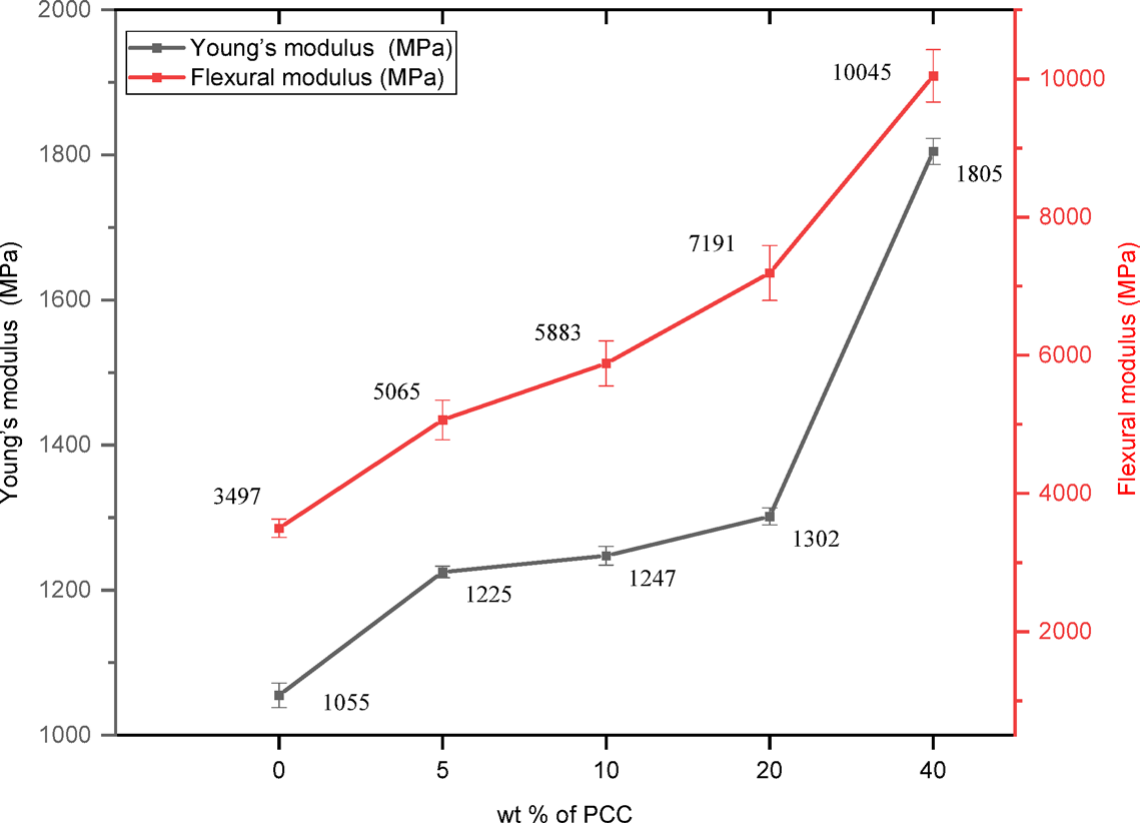
Young’s modulus and flexural modulus of PVC/PCC compounds.

Figure 8 illustrates how the addition of PCC enhanced the PVC/PCC compounds’ flexural modulus and Young’s modulus. The two main causes of the increase in elastic modulus are the substitution of rigid particulate fillers for a polymeric matrix and the rigid PCC particles’ limitation of chain mobility, which prevents deformation^25,49,50^.

**Fig. 9:**
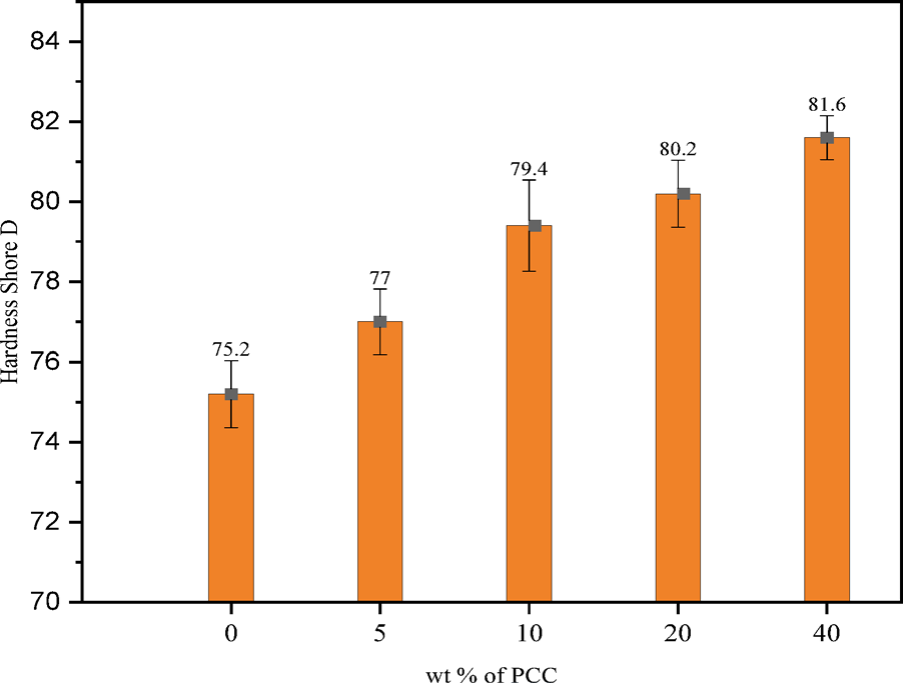
Hardness shore D for PVC/PCC compounds.

The resistance of a specimen to penetration by an indenter of a specific form under a specific load is known as shore hardness^51^. Figure 9 shows an increase in hardness with the addition of PCC. The hardness increased from 75.2 for S0 (neat PVC compound) to 77 for S5, then to 79.4 for S10, 80.2 for S20, and 81.6 for S40. The rigid character of PCC can explain this^52–54^. The results showed that PVC compounds have great resistance to indentation, and they are hard materials.

#### Physical Properties

**Fig. 10:**
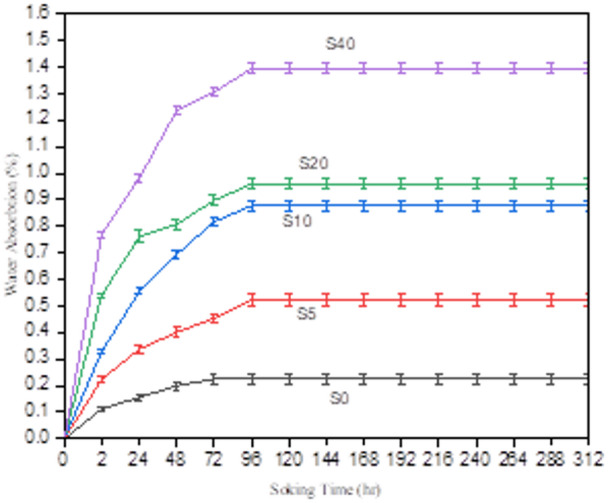
Water absorption (%) of PVC/PCC compounds at different ratios.

Water absorption curves for PVC/PCC compounds with varying PCC loading at varying immersion times are displayed in Figure 10. According to the results, filler loading and particle size affect the compounds’ water absorption. It was found that when filler loading increased, water absorption increased. Generally, incorporating PCC particles resulted in a slight increase in the compounds’ water absorption, with an almost linear trend during the initial stages. After that, there is no further water absorption by the compounds; this may be because PCC particles have little effect on water absorption beyond this saturation limit. This was expected due to their low water solubility and low water diffusion^33^. Moreover, the overall increase in water absorption upon the addition of PCC can be attributed to the porous nature of PCC particles, which, in turn, increases the compounds’ water-holding capacity^52^.

The specific gravity for PVC/PCC compounds increased linearly with increasing the content of PCC, it was increased from 1.363 (for S0) to 1.371 (0.59%), 1.391 (2.05%), 1.425 (4.5%), and 1.456 (6.8%) for S5, S10, S20, and S40, respectively. This may be because PCC has a higher density than PVC resin, suggesting that the filler particles add weight to PVC matrix^46^.

#### Thermal properties

Since PVC is considered a polymer that can change its characteristics when exposed to different temperatures, particularly higher ones, testing its thermal behavior is crucial. The sample’s thermal behavior can be predicted from the weight-temperature curve by recording its weight at a constant heating rate as the temperature increases. This method may be used to examine substances that change mass, whether through oxidation, breakdown, or the loss of volatile substances such as moisture. Commonly, nitrogen is employed as an inert/purge gas to remove evaporated sample material. By plotting the thermogravimetric curves of the samples, we can determine which sample is most thermally stable^55^.

**Fig. 11:**
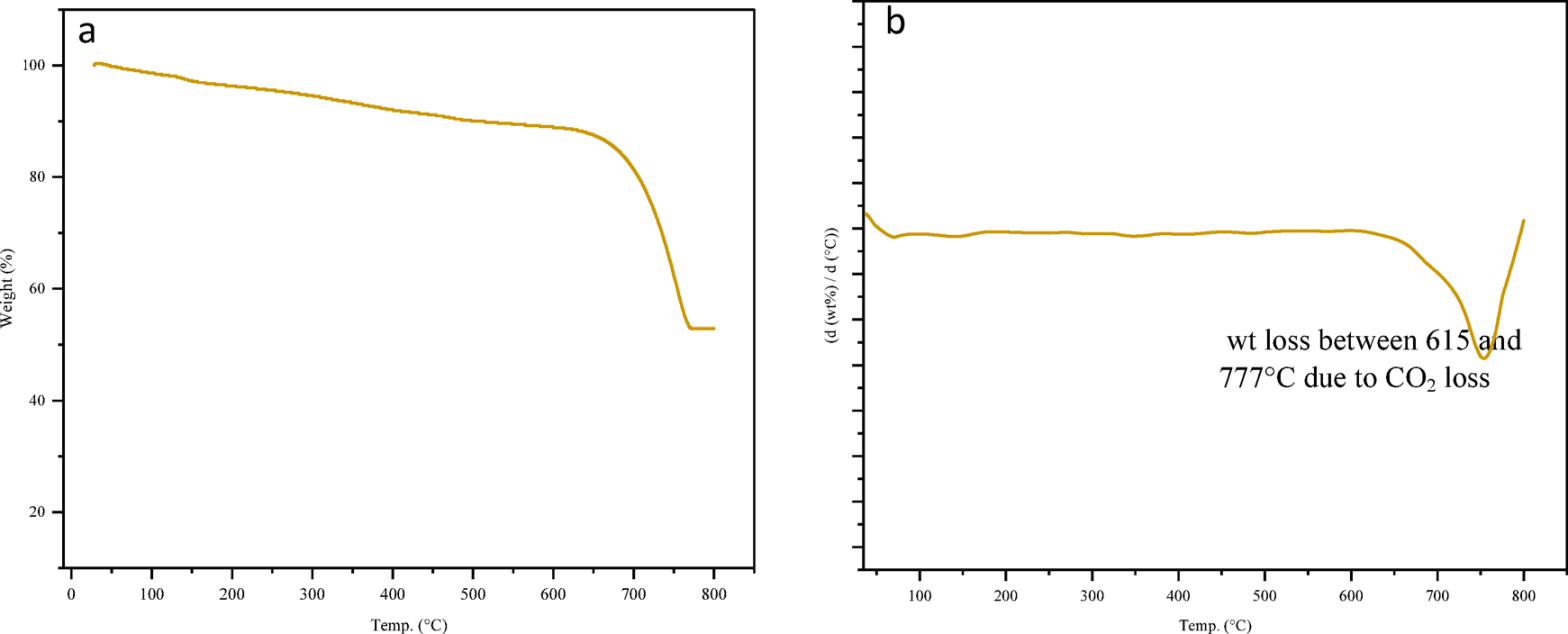
**(a)** TGA curve for PCC and **(b)** DTG curve for PCC.

The TGA and DTG curves of PCC are shown in Figure 11a & b, respectively, and explained in Table 4. First, there were three different stages of weight reduction for PCC. The initial phase of weight loss in the 25–125 °C range was around 2%; this may be due to moisture absorption. The second stage of weight loss, which occurred between 125 and 615 °C, was 9.39%. This might be because organic matter adsorbed onto PCC was lost during the beet sugar treatment^56^. With a weight loss of around 35.9%, the third stage of weight loss occurs between 615 and 777 °C. This might be because of CO2 loss in pure CC; the theoretical CO2 loss is 44%. The CO2 weight loss was 35.9% based on the TGA data. Consequently, the calcium carbonate content in PCC was 81.59%^57^. This was in accordance with the volumetric method results mentioned earlier in the characterization of PCC.

**Fig. 12:**
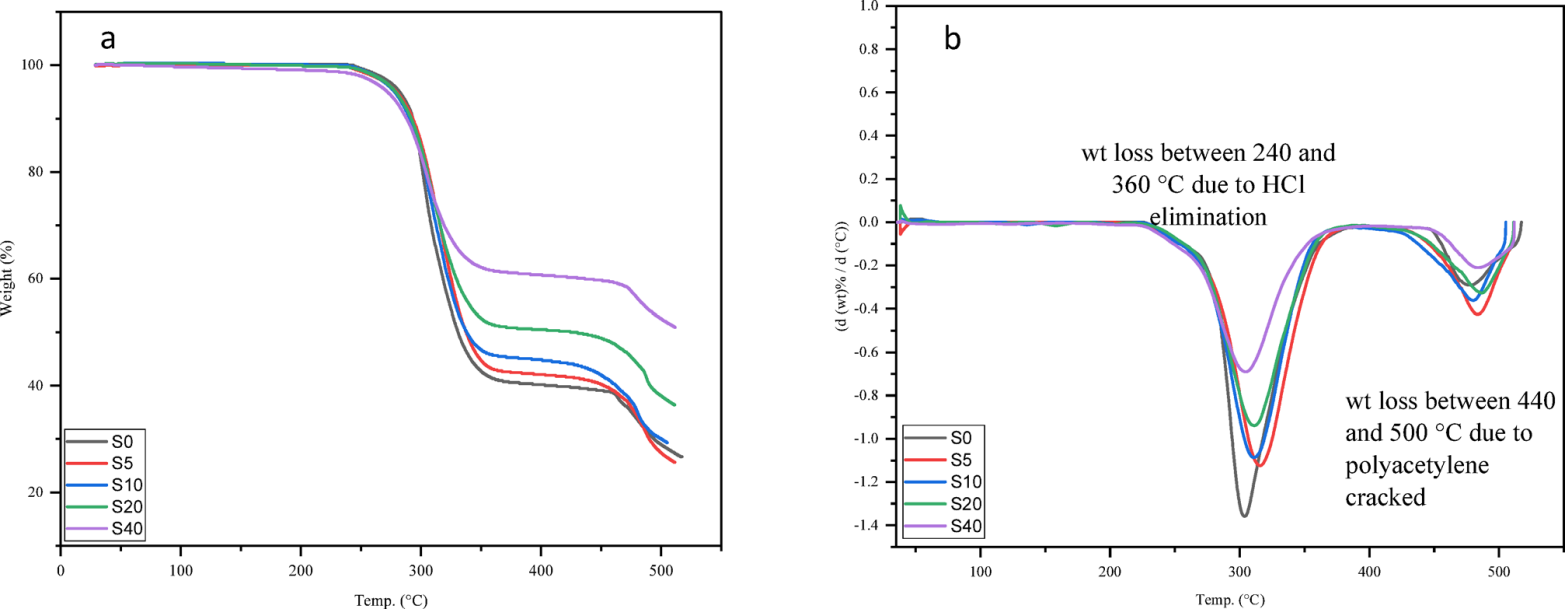
**(a)** TGA curve for S0, S5, S10, S20 and S40 and **(b)** DTG curve for S0, S5, S10, S20 and S40.

The TGA and DTG curves for S0, S5, S10, S20, and S40 are shown in Figure 12a & b, respectively, and are explained in Table 4. The TGA curve shows two distinct weight-loss stages for the neat PVC compound (S0). The initial phase of weight loss occurred between 240 and 360 °C, and the peak degradation temperature (T_peak1_) was 300 °C, mainly because of the polyene structure’s creation resulting from the hydrochloric acid (HCl) elimination process of PVC molecules; the sample weight loss in this range was (58.89%). With increasing temperature, a covalent HCl bond was created when hydrogen radicals from adjacent (C-H) groups were removed by chlorine radicals created by the scission of (C-Cl) labile bonds. As a result of this chemical process, the polymer chain began to create double bonds. Once this kind of reaction started, it spread along the chain, forming new H-Cl molecules. In the range of (360 and 440 °C), PVC compound became thermally stable, meaning the weight did not decrease. Indeed, during (HCl) release, conjugated double bonds formed, resulting in the formation of a new polymer, polyacetylene. Compared to PVC, this polymer has better thermal stability. The temperature range for the second stage of weight loss was 440–500 °C, with Tpeak2 at 464 °C; polyacetylene cracked, indicating that covalent and multiple bonds were split^58^.

**Table 4:** The decomposition stages and the weight loss for PVC/PCC compounds extracted from TGA.

Samples	First decomposition stage				Second decomposition stage			
	Start (^°^C)	End (^°^C)	T_peak1_ (^°^C)	wt % loss	Start (^°^C)	End (^°^C)	T_peak2_ (^°^C)	wt % loss
S0	240	360	300	58.89	440	500	464	12.34
S5	240	360	319	56.25	440	500	473	14.87
S10	240	360	317	53.58	440	500	475	13.79
S20	240	360	314	47.94	440	500	476	12.65
S40	240	360	308	37.00	440	500	467	8.76

However, the inclusion of PCC significantly altered the peak degradation temperatures and weight loss percentages of the PVC matrix. During the first decomposition stage, which occurred between 240 °C and 360 °C, the neat PVC (S0) exhibited a Tpeak1 of 300 °C and a weight loss of 58.89%. The addition of just 5% PCC (S5) induced a dramatic positive shift, raising Tpeak1 to 319 °C, while the weight loss decreased to 56.25%. This significant improvement was attributed to the fine particle size and high surface area of PCC, which facilitated its role as an effective acid acceptor. The well-dispersed particles created a large interfacial area with the PVC matrix, allowing them to efficiently neutralize the evolved HCl gas and retard the autocatalytic dehydrochlorination process, thereby acting as a secondary stabilizer^19,59^. The same mechanisms apply to ground calcium carbonate (GCC), but PCC performs better due to its smaller particle size and greater surface area^12^. However, as PCC loading increased to 10% (S10) and 20% (S20), T_peak1_ decreased gradually to 317 °C and 314 °C, respectively, culminating in a drop to 308 °C at 40% loading (S40).

This decrease in peak temperature at higher loadings was directly caused by severe particle agglomeration. As the PCC content exceeded optimal limits, the particles clumped together, drastically reducing their effective surface area and hindering their ability to scavenge the evolved HCl gas. Furthermore, these large agglomerates formed a poor interfacial connection with the PVC matrix, resulting in microscopic voids within the composite structure^16,17,19^. Concurrently, the weight loss in this first stage continued to drop steadily across these samples, from 53.58% to 47.94% for S10 and S20, respectively, reaching a minimum of 37% for the S40 compound. This simultaneous decrease in both T_peak1_ and weight loss reveals a critical contrast. The drop in T_peak1_ confirms that agglomeration causes PVC to degrade sooner, likely due to localized stress concentrations at the voids. However, the steady reduction in weight loss was due to the mass-dilution effect. As the thermally stable inorganic PCC replaced PVC matrix on a per-unit-mass basis in the composite, less organic material was available to decompose during this first stage.

A similar phenomenon was observed during the second decomposition stage, which occurred between 440 °C and 500 °C, with a T_peak2_ of 464 °C and a weight loss of 12.34% for S0. T_peak2_ steadily increased with the addition of PCC, reaching a maximum of 476 °C at 20% loading (S20). The improvement in heat stability with increasing PCC content may be due to PCC’s ability to promote char formation and prevent volatile breakdown products from diffusing, indicating a robust physical barrier effect that protected the residual char^60^. However, at the extreme 40% loading (S40), severe filler agglomeration disrupted the continuity of this protective char layer, causing T_peak2_ to drop back to 467 °C.

Despite the high agglomeration of PCC particles at the 40% loading that caused both peak temperatures to decline from their optimum values, the weight loss percentages consistently decreased across both stages, falling to just 8.76% in stage 2 for S40. This continuous reduction in weight loss occurred because the high concentration of the thermally stable inorganic PCC displaced the polymer volume. Essentially, a mass-dilution effect occurred; as the filler content increased, the actual volume of PVC matrix available for decomposition decreased proportionally. This resulted in a higher residual mass and a lower total weight-loss percentage, completely independent of the compromised thermal barrier. Even with agglomeration issues, both _Tpeak1_ and T_peak2_ at 40% load of PCC remained consistently higher than those of the unfilled S0 matrix, confirming that the presence of the stable inorganic filler enhances the thermal stability of PVC/PCC compounds.

**Fig. 13:**
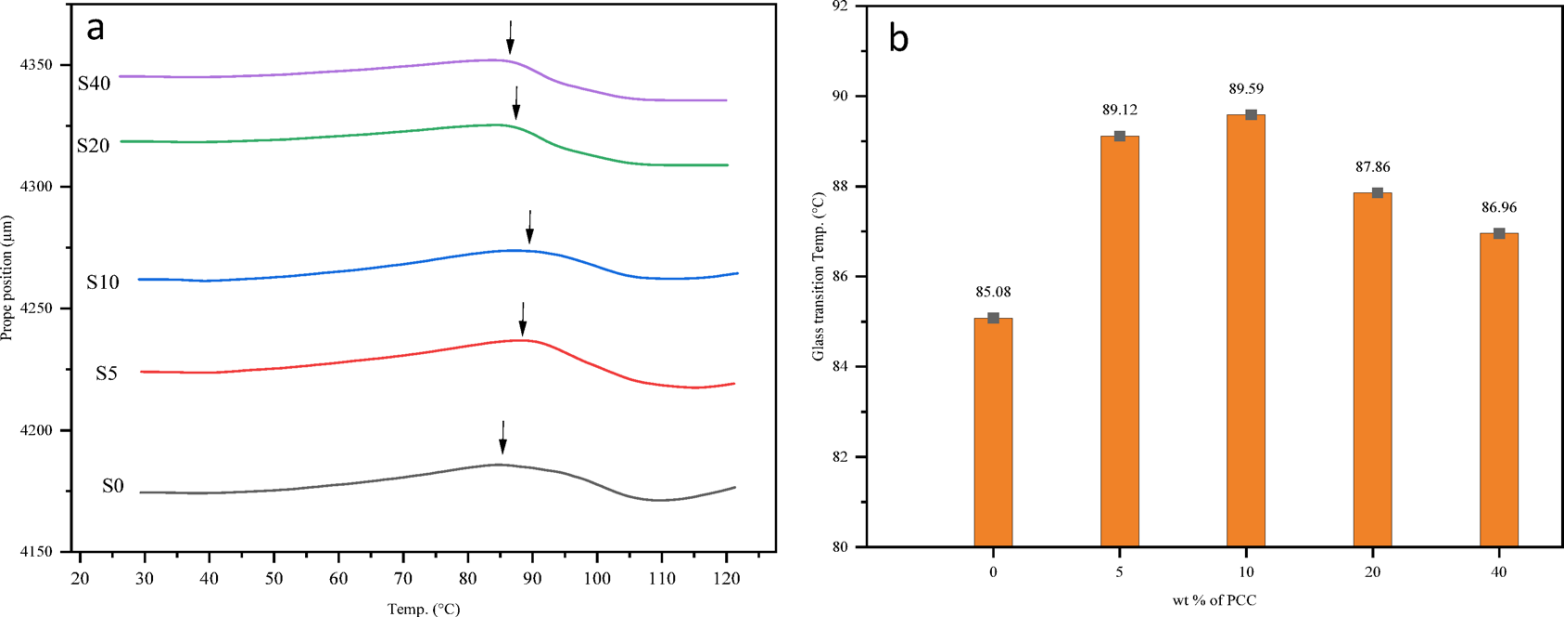
**(a)** TMA curves for S0, S5, S10, S20, and S40, (**b)** Effect of loading PCC to neat PVC compound on T_g_.

The glass transition temperature (T_g_) is an essential property of glass-forming materials that determines their industrial applicability and processing methods. Strong chlorine-carbon polar interactions give PVC a high T_g_. The majority of previous research has confirmed that adding mineral fillers to PVC increases T_g_, which can be explained by restricting macromolecular mobility^40,46,61^. The T_g_ of neat PVC and PVC/PCC compounds was determined by TMA. Figures 13a shows TMA curves for S0, S5, S10, S20, and S40, respectively. Figures 13b shows the summarized T_g_ values for neat PVC and PVC with PCC at different loading ratios. It can be concluded that as the PCC content increases, the T_g_ of these compounds initially increases, then decreases, but remains higher than that of neat PVC. This general increase may be due to the mobility of PVC molecular chain segments being restricted by the presence of PCC particles in PVC matrix; hence, the segments of PVC require more energy to move^26^. Moreover, the free volume theory may also be used to explain it^62^. The theory states that a solid or liquid’s volume consists of two components: the occupied volume, which comprises the molecules themselves, and the free volume, which comprises the space between them. The latter is what allows molecular segments to move. The free volume remains constant below T_g_, but increases above T_g_, allowing molecular segments to adjust their conformation within the available space. The addition of PCC particles in PVC resulted in a decrease in the free volume inside PVC matrix. Therefore, it needed a greater amount of energy to expand the free volume and then maintain the molecular segments’ movement. This ultimately led to a higher T_g_ for the free volume, thereby maintaining molecular chain motion. However, beyond 20 wt%, the decrease in T_g_ correlates with PCC agglomeration (as shown in SEM). Because of their low interfacial adhesion, the agglomerates act as defects. This results in two effects: (1) stress concentration at the particle-matrix interface, and (2) the creation of mobile polymer chains that are poorly adhered to the filler surface. All of these factors worked together to increase the free volume around the agglomerates, promoting earlier chain mobility and lowering the glass transition temperature.

## Conclusion

The major reasons for using the vast volumes of PCC waste from the sugar beet process as a filler in PVC compounds are a growing awareness of environmental issues and lower raw material costs. Compared with a neat PVC compound, this compound should be less expensive and exhibit higher Young’s modulus, flexural modulus, flexural strength, thermal stability, and glass transition temperature. Because PCC is harder than neat PVC compound, the hardness Shore-D improved when PCC was added. Young’s modulus and flexural modulus of compounds were enhanced by increasing PCC loading. The flexural strength of the compounds was improved by adding PCC; the maximum value was observed at 5 wt% PCC (35% improvement over the neat PVC compound). As the PCC content increased, the tensile strength and elongation at break of the compounds decreased. The morphology of the tensile cracked surfaces of PVC/PCC compounds was examined using SEM. It is evident that PCC particles are more evenly dispersed throughout the PVC matrix at lower PCC contents; however, as PCC content exceeds 20 wt%, some particles agglomerate. PVC/PCC compounds’ water absorption was slightly larger than that of the neat PVC compound because of the porosity inside PVC chains created by PCC. The specific gravity of PVC/PCC compounds was greater than that of the neat PVC compound because PCC has a higher density than PVC resin. Because PCC is more thermally stable than PVC, its smaller particle size, distributed within the polymer chains, increases the overall surface area of contact between the PVC matrix and PCC particles, thereby protecting PVC from thermal degradation. Additionally, PCC may neutralize HCl, acting as a secondary thermal stabilizer for PVC, so the PVC/PCC compound was more thermally stable than the neat PVC compound. Finally, because PCC particles in the polymer matrix inhibit the mobility of PVC chain segments, the glass transition temperature (T_g_) of the PVC/PCC compounds was higher than that of neat PVC. This means that the polymer chain segments needed more energy to move.

## Data Availability

All data generated or analyzed during this study are included in this published article.
